# Implicit preference towards slim bodies and weight-stigma modulate the understanding of observed familiar actions

**DOI:** 10.1007/s00426-018-1030-7

**Published:** 2018-06-08

**Authors:** Valentina Cazzato, Stergios Makris

**Affiliations:** 1 Division of Psychology, University of Bradford, Bradford, UK; 2 School of Natural Sciences and Psychology, Liverpool John Moores University, Tom Reilly Building, Byrom Street, Liverpool, L3 3AF UK; 3 Department of Psychology, Edge Hill University, Ormskirk, Lancashire, L39 4QP UK

## Abstract

Mounting research evidence suggests that motor resonance (MR, i.e., the mapping of others’ actions onto one’s own motor repertoire) can be influenced by diverse factors related to individual differences. However, no evidence has been reported so far on the effects of physical appearance and negative attitudes toward obesity to the mechanism of MR. Thirty-six participants (18 normal-weight and 18 overweight) performed a weight discrimination task, in which they were observing amateur actors reaching and grasping a light or heavy cube with or without deception (true vs. fake actions). At the end of each video clip, participants were instructed to indicate the correct cube size (light or heavy). Importantly, body similarity between observers and actors was manipulated by presenting videos of normal-weight or overweight actors. Fat phobic attitudes and automatic preference for normal-weight than obese people were also examined. Signal detection analysis (*d*′) on the acquired accuracy data has shown that both normal- and overweight participants were able to better discriminate truthful actions when performed by the normal-weight as compared to overweight actors. Furthermore, this finding was negatively correlated with increased scores of fat phobic attitudes in both groups. Hence, for the first time, we provide experimental evidence of action simulation being modulated by an implicit visual sensitivity towards slim bodies.

## Introduction

Motor resonance (MR) refers to the phenomenon in which when one person observes another performing a motor act, this perception automatically activates a similar network of brain regions that would be involved during action execution. Recent studies have shown that MR activity in the brain is modulated by the specific observer’s motor repertoire, determined by their specie-specific behaviours (Buccino et al., 2001, 2004), cultural learning (Molnar-Szakacs, Wu, Robles, & Iacoboni, 2007), motor expertise (Abreu et al., 2012), ethnicity (Müller et al., 2011), and individual differences in personality traits, such as narcissism (Hogeveen & Obhi, 2013; Obhi, Hogeveen, Giacomin, & Jordan, 2014) or power (Hogeveen, Inzlicht, & Obhi, 2014). Furthermore, recent studies on pain perception (Avenanti, Sirigu, & Aglioti, 2010; Azevedo, Macaluso, Avenanti, Santangelo, Cazzato, & Aglioti, 2013; Azevedo, Macaluso, Viola, Sani, & Aglioti, 2014) have reported the influence of physical similarity and group membership on the empathic resonant neural responses to others’ pain. Hence, the observation of conspecifics may influence our bodily perceptions and actions by means of creating an anticipatory representation of others’ actions in relation to physical and psychological similarities between the observer and the actor.

Moreover, the visual perception of bodies not only can inform us about someone’s intentions, but can also be critical for successful social interaction between humans. For example, it has been shown that the pervasive tendency towards the body thinness ideal documented in Western Societies (Thompson & Stice, 2001) can lead to negative prejudice towards obese individuals (i.e., anti-fat bias), which is reciprocally linked to the empathic abilities to understand their emotions and intentions (Gapinski, Schwartz, & Brownell, 2006). Despite the ‘explicit’ evidence reported by several studies on negative attitudes towards obese, other studies attempted to investigate the ‘implicit’ core of this stigmatizing behaviour. For example, a recent study by Schupp and Renner (2011) aiming at exploring the implicit nature of anti-fat bias by means of event-related brain potential recordings (ERP) suggested that the anti-fat bias may occur as a reflex, as well as independent of explicit processing goals (Schupp & Renner, 2011). The importance of revealing the implicit mechanisms of this anti-fat bias resides in offering a unique understanding of the unconscious processing that may affect not only real-life social interactions, but also the motor resonance mechanism.

To the best of our knowledge, evidence of whether body similarity as conveyed by body weight may affect the ability to simulate observed ongoing actions is still elusive. Hence, in the present study, we aimed at investigating whether simulating others’ actions may be affected by the correspondence between the body weight of the observer and that of the actor. To this aim, we conducted a study implementing a behavioural implicit weight discrimination task (WDT, Finisguerra, Amoruso, Makris, & Urgesi, 2018) along with a crucial manipulation of physical similarity between the observer and the actor as conveyed by body weight. Perceptual weight judgments have been proposed to involve the mapping of a perceptual representation of the observed actions onto a representation of the appropriate motor pattern for the same actions in the observer (e.g., Valchev et al., 2015, 2017; Podda, Ansuini, Vastano, Cavallo, & Becchio, 2017; Alaerts et al., 2010; Bosbach, Cole, & Prinz, 2005).

Furthermore, the perception and simulation of others’ actions may also include another social aspect, which is the understanding of the actor’s intentions. Indeed, interpersonal interactions may require relying on action observation and simulation to understand whether another person is honest or deceitful. With regards to the simulation of bluffing actions, there is some initial causative evidence showing that deceptive action intentions and movement kinematics, as compared to truthful ones, may be coded by the observer’s motor system at different hierarchical levels of action representation (Tidoni, Borgomaneri, Di Pellegrino, & Avenanti, 2013; Finisguerra et al., 2018). Following up from this evidence, we have also added in our task two different types of action (truthful vs. fake) and we expected MR, as measured by the performance in the task (i.e., perceptual discrimination, *d*′), to be differently modulated by these two conditions. Finally, as people are more likely to prefer normal-weight to obese people, we predicted the degree to which motor resonance is susceptible to top–down regulation (i.e., is reduced in the deceptive than truthful movement condition) and would vary as a function of prejudice towards (out-group) obese individuals, as measured by a weight-implicit association test (IAT) and Fat Phobia scores.

## Methods

### Participants

A total of 36 students (18 normal-weight, 11 women; 18 overweight participants, 12 women) from the University of Bradford participated in the experiment. Participants were recruited on the basis of their body mass index (BMI). According to the World Health Organisation (WHO, 2000), participants with a BMI comprised between: 18.5–25 kg/m^2^ were classified as normal-weight, while participants with a BMI above 25 kg/m^2^ were considered overweight/obese. Participants’ BMI was obtained from measuring weight (kg) and height (cm), by means of a digital scale (Tanita Body Composition Analyser BC-420MA). Participants were naïve as to the purposes of the experiment and information about the experimental hypothesis was provided only after the experimental tests were completed. All subjects, but one male, were right-handed as ascertained by means of a Standard Handedness Inventory (Briggs & Nebes, 1975). They were native English speakers of Caucasian and South-Asian ethnicity. All participants reported normal or corrected-to-normal vision; all were in good health, free of psychotropic or vasoactive medication, with no past history of psychiatric or neurological disease. At the end of the experiment, participants filled the following self-report questionnaires: (1) the Fat Phobia Scale—short form (Bacon, Scheltema, & Robinson, 2001), which assessed explicit negative attitudes and stereotyped perceptions of obese people. In this measure, 14 pairs of adjectives are used to describe obese people (e.g., “lazy” vs “industrious”, “no will power” vs “has will power”), and respondents are asked to indicate on a scale from 1 to 5 which adjective they feel best describes their beliefs about obese people. A score of 2.5 indicates neutral attitudes about obese persons, with scores more than 2.5 reflecting higher levels of fat phobia (more negative attitudes) and lower scores indicating more positive attitudes; (2) The Weight-Implicit Association Test (weight-IAT, Greenwald, Nosek, & Banaji, 2003), which has proven to be a useful tool in the study of prejudice towards several social groups (Nosek, Hawkins, & Frazier, 2011), including obese individuals (Bessenoff & Sherman, 2000; Schwartz, Chambliss, Brownell, Blair, & Billington, 2003; Teachman, Gapinski, Brownell, Rawlins, & Jeyaram, 2003; O'brien, Puhl, Latner, Mir, & Hunter, 2010). This test provides a measure of an automatic preference for normal-weight than obese people as a weight-related implicit attitude.

The participants’ demographics and self-report questionnaire scores as a function of group are reported in Table 1. As expected, an independent sample *t* test indicated that BMI was significantly higher in overweight than in normal-weight participants. However, the two groups were matched for age, Fat Phobia, and weight-IAT scores. Participants gave their written informed consent, and the procedures were approved by the University of Bradford ethics committee and were in accordance with the ethical standards of the 1964 Declaration of Helsinki.

**Table 1:** Mean and standard error of the mean (SEM, in brackets) of participants’ demographic information as a function of groups’ weight (normal-weight vs. overweight)

	Normal-weight *n* = 18	Overweight *n* = 18	Normal-weight vs. overweight
Age	25.28 (0.99)	24 (0.89)	*t*(34) = 0.96; *p* = 0.344
BMI	22.24 (0.43)	31.98 (1.19)	*t*(34) = − 7.69; *p* < 0.001
Weight-IAT	0.53 (0.08)	0.38 (0.10)	*t*(34) = 1.19; *p* = 0.243
Fat Phobia Scale	2.96 (0.14)	3.25 (0.19)	*t*(34) = − 1.21; *p* = 0.235

The data of participants were compared by means of independent sample *t* test

*BMI* body mass index, *Weight-IAT* weight-implicit association test (*D* score)

### Weight discrimination task stimuli

Stimuli for the WDT consisted of a series of video clips depicting a normal- or overweight amateur model (2 males or 2 females respectively per each body weight) while performing a reaching, grasping, and lifting action towards a metal cube. Two types of metal cubes were used. They were both identical in size (approximately 5 × 5 × 5 cm), but with different weights (approximately 100 and 800 g, respectively). The videos were recorded from the posterior plane, depicting only the model’s right arm, the cube, and the placing box. During the first part of the recording, the experimenter correctly informed the models about the actual weight of the cube and asked them to perform a congruent action (true actions, TA). In the second part of the recording, they were again informed about the true weight of the cube, but, this time, they were instructed to perform an incongruent action; that is to pretend that the cube was light for the heavy one and vice versa for the light cube (fake action; FA) (Tidoni et al., 2013; Finisguerra et al., 2018). Before starting the recording, the actors were briefly trained to perform the movement. For each of the four actors, four types of videos were created following a 2 (cube weight: light, heavy) × 2 (actions type: TA, FA) design: light TA, heavy TA, light FA, heavy FA. This way, 16 videos were created in total.

The video clips were recorded with a Canon HD camera (Canon LEGRIA HF R77). Each video clip was edited to have the same duration of 1600 ms and then split in 8 frames (200 ms, presented at 5 Hz) using Adobe Premiere Software (Adobe Systems Incorporated, San Jose, CA). Before that, the original video recordings were transformed to black and white video clips to prevent local changes in skin tone during hand contraction from revealing information about the real weight of the cubes. Furthermore, videos were carefully checked for the absence of local hand information (e.g., hair).

Importantly, a kinematic analysis was performed (see ‘Stimuli kinematic analysis’ section) to control for differences in actions’ kinematics as performed by the two models’ weight and to make sure that video clips contained subtle movement cues that could be used to detect models’ intent to deceive while performing the WDT.

Finally, at the end of the experimental session, we asked participants to position the computer mouse along a 100-mm visual analogue scale (VAS) to provide a series of judgments for each model. In particular, the participants were requested to report: ‘How much overweight does this person look to you?’ on a scale from 0 (not overweight at all) to 100 (very overweight). The analysis of the overweight VAS scale confirmed the success of our models’ weight manipulation, being the overweight models judged more overweight than the normal-weight models (56.81 ± 3.04 vs. 26.64 ± 2.51, *t*(35) = 12.10, *p* < 0.001). Importantly, no statistical differences were observed for overweight (55.14 ± 4.77 vs. 58.47 ± 3.87, *t*(34) = − 0.54, n.s.) and normal-weight models (24.56 ± 4.07 vs. 28.72 ± 2.99, *t*(34) = − 0.83, n.s.) among the two groups.

During the experiment, all participants were seated in a dimly lighted room in front of a 19-inch LCD monitor (resolution 1027 × 768 pixels, screen refresh frequency at 60 Hz), in which videos were presented on a black background, subtending a 14.5 × 11.5 region of optical view.

### Kinematic analysis of the WDT stimuli

**Fig. 1:**
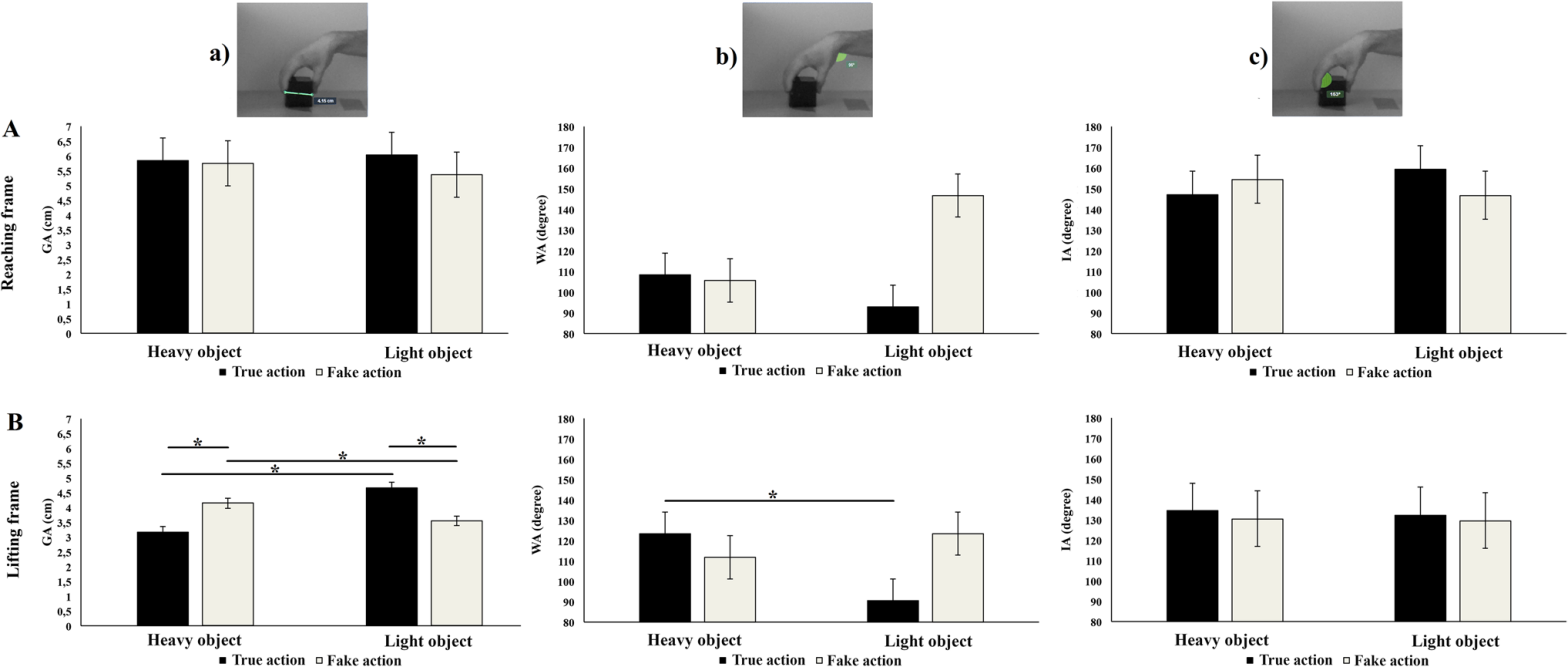
Spatial kinematic parameters (mean ± SEM) during the reaching (**A**) and the lifting (**B**) phase of true and fake actions performed by normal- and overweight models upon the light or the heavy object. Asterisks indicate significant comparisons (*p* < 0.05). Pictures on the top represent: (**a**) the grip aperture (GA, in cm) parameter, which is measured taking into account the distance between the tips of the thumb and of the index finger; (**b**) the wrist aperture (WA, in degrees) parameter, measured at the palmar side of the radiocarpal joint and was defined by the line connecting the lateral epicondyle of the humerus with the radial styloid process and the line connecting the thumb metacarpal joint with the radial styloid process, and (**c**) the index angle (IA, in degrees) parameter, measured by the line connecting the distal with the proximal interphalangeal joint of the index finger and the line connecting the index finger metacarpal joint with the proximal interphalangeal joint of the index finger

To identify whether true and fake actions differed in their kinematics and to exclude confounding factors linked to different kinematics of the actions performed by the two models’ weight, we analysed three spatial parameters of the models’ right arm while performing the true and deceptive actions, using a dedicated software for motion kinematics analysis (Kinovea 0.8.15). Specifically, grip aperture (GA), wrist angle (WA), and index angle (IA) were computed at two frames: 200 ms before (reaching phase) and 200 ms after the hand-object contact point (Finisguerra et al., 2018). The GA was calculated as the distance between the tips of the thumb and the index finger (in cm). The WA was measured at the palmar side of the radiocarpal joint, and was defined by the line connecting the lateral epicondyle of the humerus with the radial styloid process and the line connecting the thumb metacarpal joint with the radial styloid process. The proximal interphalangeal IA was measured by the line connecting the distal with the proximal interphalangeal joint of the index finger and the line connecting the index finger metacarpal joint with the proximal interphalangeal joint of the index finger. The latest two measures were expressed in degrees (see Fig. 1).

### Weight discrimination task

**Fig. 2:**
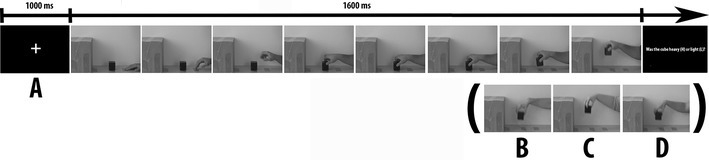
Sequence of presentation in a typical trial for the weight discrimination task. Pictures represent video-clip frames during which (**a**) an overweight male model performs a true action, (**b**) a normal-weight male model performs a true action, (**c**) an overweight female model performs a true action, and (**d**) a normal-weight female model performs a true action

The experiment was created with E-Prime software (version 2.0, Psychology Software Tools, Inc., Pittsburgh, PA) and it consisted initially of the request for the participants’ demographic details, followed by brief written task instructions and, finally, by the self-report questionnaires and rating scale of the models’ weight. Each trial started with the appearance of a black central fixation-cross presented on a grey light background. After 1000 ms, the video depicting a normal- or overweight model (male or female) performing a true or fake action appeared for 1600 ms at the centre of the screen, subtending a visual angle of approximately 12° × 10° (see Fig. 2). Participants completed an 8-trial practice block before proceeding to the experimental blocks. During the experimental session, three blocks of 32 trials each were presented. Each participant was tested in a single experimental session lasting about 1 h.

### Weight-IAT

**Fig. 3:**
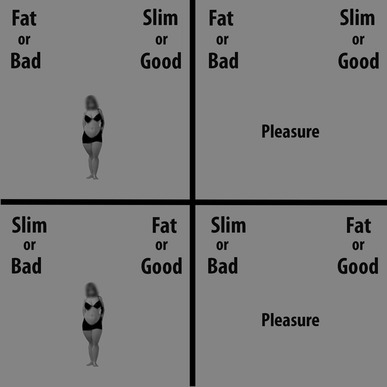
Example of the weight-IAT. Typically, participants are faster and more accurate when ‘Good’ shares a key with a slim person or a good word and ‘Bad’ with an obese person or a bad word (in the congruent-stereotype blocks, top) than when the pairings are switched (in the incongruent-stereotype blocks, bottom). This demonstrates an automatic association between ‘Fat’ and ‘Bad’ categories and ‘Slim’ and ‘Good’ categories

The weight-IAT was created with E-Prime software (version 2.0 Professional, Psychology Software Tools, Inc., Pittsburgh, PA). Participants were required to answer as fast and accurately as possible after the onset of the stimuli (i.e., single words or images presented one at a time at the centre of the screen), by pressing a left (E) or a right (I) key on a computer keyboard with the index finger of the left hand and right hand, respectively. The IAT lasted approximately 8 min and was administered in seven blocks, consisting of both congruent and incongruent conditions (blocks 3, 4, 6, and 7) and familiarization blocks (blocks 1, 2, and 5) (Greenwald, Nosek, & Banaji, 2003; Cazzato, Makris, & Urgesi, 2017). Before the first presentation of the weight-IAT, participants were shown a list with all the words belonging to the two relevant categories and were asked to carefully read all the stimuli. In the first block of the weight-IAT, 12 images of Obese and 12 images of Slim people were presented and had to be classified as being either ‘Fat’ (left key) or ‘Slim’ (right key) (Cazzato, Siega, & Urgesi, 2012). Each of the 12 images of the two categories was presented only once for a total of 24 trials. The second block also consisted of 24 trials in which Bad-related (requiring a left-key response) and Good-related (requiring a right-key response) words were presented. In the third block (24 trials practice) and in the fourth block (48 trials test), both Obese and Slim bodies and Good and Bad words were randomly presented and participants were instructed to press the left key for Bad-related words and images of Fat people, and the right key for Good-related words and images of Slim people (congruent-stereotype condition). In the fifth block (24 trials), response key assignments were reversed in relation to the categorization involving images of fat people (right-key) and images of slim people (left-key). Finally, in the sixth block (24 trials practice) and in the seventh block (48 trials test), both Obese and Slim bodies and Good and Bad words were randomly presented and participants were required to press the left key for images of Obese people and Good words and the right key for images of Slim people and Bad words (incongruent-stereotype condition) (see Fig. 3). Typically, participants are faster and more accurate in the congruent- than in the incongruent-stereotype blocks, thus demonstrating an automatic association between Obese and Bad categories and Slim and Good categories (Greenwald, Nosek, & Banaji, 2003). Stimuli were randomly presented within each block. Each word/image remained on the computer screen until the participant gave a correct response in each trial. Indeed, if an error occurred in a trial, a red “X” appearing below the word stimulus prompted the participant to correct the mistake by pressing the correct key. Following the response, the next stimulus appeared after 500 ms, during which only the category labels were visible on the screen. The response latency data for each participant were transformed into *D* scores using the *D*-algorithm, as developed by Greenwald, Nosek, & Banaji, (2003). Accordingly, a positive *D* score indicates that a participant responded more quickly when categorizing positive adjectives with ‘Slim’ and negative with ‘Fat’, than when categorizing in the opposite manner (‘Slim’ with negative and ‘Fat’ with positive).

### Behavioural data handling

Behavioural performance obtained at the two-alternative-forced-choice WDT was analysed using the signal detection theory (SDT). Based on SDT, we calculated *d*′ as a measure of (perceptual) sensitivity and *β* as a measure for the likelihood ratio or response bias (natural logarithm of *β*; ln*β*). The percentage of correct responses (accuracy) was first calculated for each participant in each experimental condition. The *d*′ and ln*β* scores were then calculated, considering whether subjects’ responses were congruent or not to the real weight of the cube. More specifically, for all true action trials, the proportion of congruent responses was considered as hits and the proportion of incongruent responses in the respective deceptive trials as false alarms. Similarly, for all deceptive trials, the proportion of congruent responses was considered as hits and the proportion of incongruent responses in the respective true trials as false alarms. This way there were separate calculations of the *d*′ and ln*β* scores for the different types of action (true, fake), as well as the two different model weights (normal-weight, overweight). For the sake of clarity, error rates of correct responses are reported in Table 2. Statistical analyses were run with STATISTICA 8.0 (StatSoft Inc, Tulsa, Oklahoma). d′ and ln*β* scores data from the WDT were entered into a mixed three-way 2 × 2 × 2 ANOVA with: 2 (actions type: TA, FA) × 2 (model’s weight: normal-weight, overweight) as within-subject factors and 2 (groups’ weight: normal-weight, overweight) as between-subject factor. The source of all significant repeated-measure ANOVA interactions was analysed using the Newman–Keuls post-hoc test. A significance threshold of *p* < 0.05 was set for all effects. Effect sizes were estimated using the partial eta-squared measure ($\eta _{{\text{p}}}^{2}$). All data are reported as mean (*M*) and Standard error of the mean (SEM). Furthermore, Pearson correlation coefficients were computed to investigate the relationship between the degree of MR effects on behavioural dependent measures and individual scores at Fat Phobia Scale and weight-IAT scores.

**Table 2:** Mean and standard error of the mean (SEM, in brackets) of participants’ accuracy as a function of Groups’ weight (normal vs. overweight), Cubes’ weight (heavy, light), Actions’ type (fake, true), and Models’ weight (normal-weight, overweight)

	Normal-weight *n* = 18				Overweight *n* = 18			
	Heavy		Light		Heavy		Light	
	Fake	True	Fake	True	Fake	True	Fake	True
Normal-weight	0.15 (0.04)	0.70 (0.05)	0.28 (0.05)	0.79 (0.05)	0.12 (0.04)	0.81 (0.05)	0.18 (0.05)	0.86 (0.05)
Overweight	0.45 (0.07)	0.61 (0.05)	0.46 (0.06)	0.73 (0.05)	0.39 (0.07)	0.61 (0.05)	0.39 (0.06)	0.80 (0.05)

## Results

### Action kinematics data

Grip aperture (GA), wrist angle (WA), and index flexion (IF) kinematics data from both the reaching and lifting phases were entered into separate factorial ANOVAs with actions type (TA, FA), models’ weight (normal-weight, overweight), and object weight (heavy, light) as between-movies factors. The results have indicated that for the reaching phases, there were no significant main effects or interactions for any of the displacement indices (see Fig. 1A).

For the lifting phase, with regards to the GA displacement, the ANOVA revealed main effects of models’ weight and object weight(*F*s > 15.082, *p* < 0.005, $\eta _{{\text{p}}}^{2}$ > 0.653), which were further corroborated by a significant two-way interaction of Models’ weight × object weight (*F*_(1,34)_ = 11.647; *p* = 0.009; $\eta _{{\text{p}}}^{2}$ = 0.593). Newman–Keuls post-hoc comparison showed that when the heavy cube was lifted by the overweight models, GA displacement was significantly smaller as compared to when lifted by the normal-weight model (overweight: 3.21 ± 0.12 vs. normal-weight: 4.10 ± 0.12, *p* = 0.002). No difference was observed between the models’ weight for the light cube (overweight: 4.06° ± 0.12 vs. normal-weight: 4.16 ± 0.12, *p* = 0.837). Furthermore, the GA for heavy cube was significantly smaller when lifted by the overweight models as compared to all conditions (all *p*s < 0.001). Importantly, the two-way interaction between object weight and action type was significant (*F*_(1,8)_ = 80.568; *p* < 0.001; $\eta _{{\text{p}}}^{2}$ = 0.910). Crucially, post-hoc comparisons showed that the lifting of the heavy object in the TA involved a smaller closure of the index finger as compared to the lifting of the same object in the FA condition (TA: 3.17 ± 0.12 vs. FA: 4.14 ± 0.12, *p* = 0.001). On the opposite, the lifting of the light object in the FA involved a smaller closure of the index finger as compared to the lifting of the same object in the TA condition (FA: 3.54 ± 0.12 vs. TA: 4.67 ± 0.12, *p* = 0.001). Thus, the displacement of the GA differentiated the truthful and the deceived conditions during lifting, but not during reaching. Importantly, no other interactions with the models weight were significant, thus demonstrating that kinematics information as conveyed by the GA displacement differentiate truthful and fake actions during lifting when performed by both normal and overweight models (*F*s < 5.000, *p*s > 0.06, $\eta _{{\text{p}}}^{2}$ < 0.385).

With regards to the WA displacement, the kinematic analysis of the data revealed only a significant two-way interaction between action type and object weight (*F*_(1,8)_ = 8.772, *p* = 0.018, $\eta _{{\text{p}}}^{2}$ = 0.523). Crucially, post-hoc comparisons showed that WA displacement for TA was bigger for the heavy object as compared to the light one (heavy: 123.25 ± 7.51 vs. light: 90.50 ± 7.510, *p* = 0.36), while no difference was observed between the two object weights for the FA condition (heavy: 111.75 ± 7.51 vs. light: 123.50 ± 7.510, *p* = 0.537). Finally, the difference between TA and FA for the light object was not significant with the WA displacement being bigger for the FA as compared to the TA ones (FA: 123.50 ± 7.51 vs. TA: 90.50 ± 7.510, *p* > 0.06). Importantly, no other interactions with the models’ weight were significant, thus demonstrating that kinematics information as conveyed by the WA displacement could be used to differentiate truthful and fake actions during lifting when performed by both normal- and overweight models (*F*s < 5.272, *p*s > 0.06, $\eta _{{\text{p}}}^{2}$ < 0.397).

Finally, concerning the IA displacement, there were no significant main effects or interactions for the IA data of the lifting frame (*F*s < 0.717, *p*s > 0.422, $\eta _{{\text{p}}}^{2}$ < 0.082). Hence, the displacement of the IA did not differentiate the truthful and the deceived conditions during the lifting phase (see Fig. 1B).

### Behavioural performance during the WDT

**Fig. 4:**
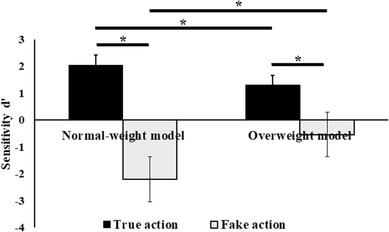
Mean (± SEM) of participants’ task sensitivity (*d*′) during observation of fake (white bars) and true (black bars) actions performed by normal- and overweight models upon the light or the heavy object. Asterisks indicate significant comparisons (*p* < 0.05)

The 2 × 2 × 2 ANOVA on the mean *d*′ scores revealed main effects of models’ weight and action type (*F*s > 26.401.916, *p* < 0.001, $\eta _{{\text{p}}}^{2}$ > 0.437), which were further corroborated by a significant two-way interaction of Models’ weight × action type (*F*_(1,34)_ = 47.239; *p* < 0.001; $\eta _{{\text{p}}}^{2}$ = 0.581). Newman–Keuls post-hoc comparisons showed that higher *d*′ scores were found when a TA, relative to a FA, was performed both by normal-weight )TA: 2.04 ± 0.21 vs. FA: − 2.20 ± 0.18, *p* < 0.001) and overweight models (TA: 1.30 ± 0.22 vs. FA: − 0.54 ± 0.25, *p* < 0.001), respectively (see Fig. 4). Furthermore, *d*′ scores for the TA performed by normal-weight models were significantly higher relative to all conditions (all *p*s < 0.005). No interactions with group were significant, suggesting that normal-weight and overweight participants did not differ in their ability to better simulate TA performed by the normal-weight as compared to overweight actors, and ruling out any spurious effects due to between-groups differences. Finally, the remaining effects were all non-significant (*F*s < 1.554, *p*s > 0.221; $\eta _{{\text{p}}}^{2}$ < 0.044).

For ln*β* scores the ANOVA revealed only a significant main effect of Action Type (*F*_(1,34)_ = 18.387, *p* > 0.001, $\eta _{{\text{p}}}^{2}$ = 0.351), with higher scores for true actions as compared to the fake ones (TA: 0.33 ± 0.08 vs. FA: − 0.25 ± 0.08). However, the effects of models’ weight on type of actions were not mediated by change in response bias since the non-significant interaction with the models’ weight, action type, and group weight (*F*s < 1.940, *p* > 0.173, $\eta _{{\text{p}}}^{2}$ < 0.054).

### Implicit and explicit anti-fat bias

One-sample *t* tests were used to compare the mean *D* scores to zero (where zero refers to the absence of any response bias) for both groups. Both normal-weight and overweight participants showed a significant stereotypical anti-fat bias, indicating that they were more prone to associate obese people to the bad-related category and normal-weight people to the good-related category than vice versa (normal-weight group: *t*(17) = 6.94, *p* < 0.001; overweight group: *t*(17) = 3.87, *p* = 0.001). Interestingly, the two groups did not differ in the levels of stereotypical anti-fat bias (see Table 1).

One-sample *t* tests were used to compare the mean Fat phobia scale scores to 2.5 (where 2.5 refers to a moderate level of explicit phobia against obese people) for each group. In accordance with implicit anti-fat bias results, both normal-weight and overweight participants showed high level of explicit negative attitudes and stereotyped perceptions of obese people (normal-weight group: *t*(17) = 3.22, *p* = 0.005; overweight group: *t*(17) = 3.89, *p* = 0.001). Furthermore, the two groups did not differ in the levels of explicit stereotypical fat phobia (see Table 1).

### Correlations with self-report measures

**Fig. 5:**
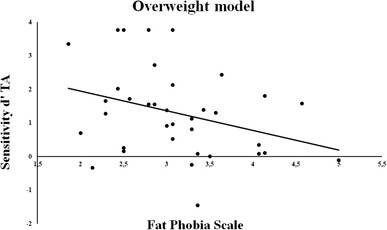
Multiple scatter plot of participants’ task sensitivity (*d*′) for the observation of True actions (TA) performed by overweight models vs. the Fat Phobia scale scores. A negative correlation indicates that participants with higher levels of fat phobia are worse at discriminating TA when performed by overweight models

Interestingly, scores on the Fat Phobia Scale were negatively correlated with *d*′ scores for TA when performed by overweight models, indicating that participants with higher (explicit) levels of fat phobia were worse at discriminating TA when performed by overweight models only (*r* = − 0.33, *p* = 0.05, see Fig. 5). On the opposite, no correlations were found between the *d*′ scores and implicit weight bias (weight-IAT) for any of the models (− 0.12 < all *r*s < 0.07).

## Discussion

The statistical analysis of the WDT data has indicated that all our participants performed better for truthful actions as compared to fake ones. This is in accordance with the previous findings reported in the literature (Tidoni et al., 2013; Finisguerra et al., 2018), and it is further corroborated by the differences in the action kinematics data. More specifically, the kinematics data have shown that there were significant differences in the way models grasped and lifted the different objects for the fake and true trials, but not when they reached to grasp those. This was somewhat predicted, as all models were expected to prepare and execute the same or similar reaching actions for objects that looked identical; however, their kinematics should be altered during the lifting phase depending on the weight of the object and the type of action that they were executing (Hamilton et al., 2007). This finding is crucially important, as we were expecting our participants to base their judgments about the weight of the object on the lifting phase of the action, during which the observed kinematics could critically inform about the cube’s weight.

Furthermore, it was found that both normal- and overweight participants performed better for truthful actions executed by the normal-weight models, thus indicating that they were able to perceptually discriminate those observed actions. Most importantly, this finding is corroborated by the fat phobia scale results showing that overall our subjects scored higher on explicit measures of a stereotypical anti-fat bias, indicating that higher levels of fat phobia were correlated with poor performance in trials executed by the overweight models. In contrast, perceptual sensitivity to fake and deceitful actions for the preferred normal-weight models was unrelated to prejudice. The degree of bias in motor resonance seems, therefore, to be influenced by how much people dislike the respective weight group. Thus, in agreement with Gutsell and Inzlicht’ (2010, 2013) findings, motor resonance seems, indeed, to be limited to the preferred normal-weight ‘in-group’ and more so when the perceiver is prejudiced or when the group in question is disliked. The present findings are illustrating that explicit negative attitudes towards obese people were detected in both of our groups and that this anti-fat attitudes could have hindered their ability to discriminate truthful actions performed by the overweight models by, in turn, modulating their actions’ simulation.

The previous studies on action observation have shown that humans have a unique ability in simulating observed action sequences and making predictions about their outcome (Urgesi et al., 2010; Avenanti, Paracampo, Annella, Tidoni, & Aglioti, 2018; Springer, Parkinson, & Prinz, 2013; Panasiti, Porciello, & Aglioti, 2017). This ability is largely depending on their previous experience with the action that they are observing and it can even modulate their ability to detect any bluffing actions and/or intentions, as well as mental states (Aglioti, Cesari, Romani, & Urgesi, 2008; Becchio, Koul, Ansuini, Bertone, & Cavallo, 2018; Makris & Urgesi, 2014). In our study, participants were observing everyday action sequences (reaching, grasping, and lifting an object) that could be truthful or fake in their execution from the onset. According to the motor resonance theory, we were expecting our participants to simulate the observed action kinematics and on that to base their predictions about the weight of the object. It has been previously reported that the detection of bluffing actions is inherent to the recognition of incongruent action kinematics and that can inform about the actor’s intentions (Makris & Urgesi, 2014; Tidoni et al., 2013). Indeed, the present results have shown that our participants were able to recognise truthful actions and, thus, make accurate predictions about the weight of the object; however, that ability was critically decremented by incongruent kinematics in the fake action sequences that could not be detected and, thus, allow for accurate judgments.

Moreover, in the present study, we tried to investigate how physical similarity as conveyed by body weight similarity between actor and observer can also modulate motor resonance. More specifically, we wanted to observe whether implicit action simulation can be modulated by differences in the body weight between our models and the participants. In that sense, we had both normal- and overweight models executing the actions, as well as our participants divided in two groups, depending on their BMI (normal-weight, overweight). Our data have indicated that both normal-weight and overweight models could better discriminate action sequences performed by the normal-weight models as compared to the overweight ones. This could be explained by the previous evidence reporting that overweight people show automatic ‘in-group devaluation’ (Rudman, Feinberg, & Fairchild, 2002), thus supporting the idea that weight does not show the in-group favouritism bias seen among other social groups (Rudman et al., 2002; Wang, Brownell, & Wadden, 2004). Furthermore, a recent study has reported a significant degree of anti-fat bias among people with high BMI (Schwartz, Vartanian, Nosek, & Brownell, 2006), thus not only confirming that overweight and obese people hold anti-fat attitudes to the same extent as do normal-weight individuals (Allison, Basile, & Yuker, 1991; Wang et al., 2004), but also highlighting the pervasiveness of this bias among the general population (Schwartz et al., 2006). This effect could be partially supported by the levels of prejudice and fat phobia measured in our participants. Indeed, we found a significant negative correlation between explicit prejudice (Fat Phobia Scale) and *d*′ scores for truthful actions performed by overweight models, indicating that participants with higher levels of prejudice were worse at simulating truthful actions when performed by disliked overweight models. It is interesting to note that no such correlation was found with the implicit weight-stigma (weight-IAT) measure, suggesting that these two measures are conceptually discrete constructs and capture different domains of knowledge and belief about obesity (Brewis & Wutich, 2012).

One consideration here is that we did not ask our participants about their perceived group membership. Although findings about their implicit and explicit negative attitudes towards overweight individuals and in both groups would speak against this possibility, it is still plausible that some did not identify themselves as part of the group, and thus felt freer to derogate obese people. Hence, future studies should take extra care in investigating those individuals who identify as overweight or obese instead of selecting participants solely based on their BMI. Furthermore, recent evidence points to the importance of incorporating self-perceptions in the stigmatization of others and the need to include self-acceptance, body shame, and beliefs about personal control into a social model of stereotyping of obese individuals (Himmelstein & Tomiyama, 2015). With these regards, the authors reported that greater body shame and beliefs about personal control predict increased anti-fat attitudes. However, at present, we cannot exclude that our findings may have been moderated by such negative self-perceptions believes (i.e., body shame, personal control, and perceived size) both in overweight and in normal-weight participants and future studies should clarify the modulatory effects of these factors. Finally, there is some initial evidence that gender differences between the actor and observer can modulate the motor resonance mechanism (Cheng et al., 2009; Lugli, Obertis, & Borghi, 2017). In the present paper, we did not investigate for such differences, as the main focus of our study remained on the modulation of the motor resonance mechanism by body appearance stereotypes, as well as the presentation of truthful vs. bluffing actions. We appreciate though that gender differences might have an effect and future studies on the topic should try to investigate further this issue.

In conclusion, the present study investigated how body differences (i.e., body weight) between actor and observer can modulate the simulation of a simple everyday action with and without deception. The present findings are in accordance with the previous studies, showing that humans have a superior ability in simulating observed actions and using this information for making appropriate judgments. Moreover, incongruent kinematics can bluff subjects with regards to the simulation of observed actions. Most importantly, we are showing for the first time to our knowledge that MR can be modulated by differences in the body weight between actor and observer that could be due to explicit fat stereotypes. This is a critical observation for better understanding the underpinnings of motor resonance in humans, as well as the mechanism of stereotype existence, such as the anti-fat bias. Further research on the topic is deemed as necessary to validate the present findings and expand research on a highly topic within social cognition and neuroscience.
